# TREM-2 Receptor Expression Increases with 25(OH)D Vitamin Serum Levels in Patients with Pulmonary Sarcoidosis

**DOI:** 10.1155/2015/181986

**Published:** 2015-06-16

**Authors:** Maria Bucova, Magda Suchankova, Elena Tibenska, Eva Tedlova, Juraj Demian, Ivan Majer, Helena Novosadova, Miroslav Tedla

**Affiliations:** ^1^Institute of Immunology, Faculty of Medicine, Comenius University, 81372 Bratislava, Slovakia; ^2^Medirex Ltd., 82016 Bratislava, Slovakia; ^3^Department of Pneumology and Phthisiology, Faculty of Medicine, Comenius University, 82101 Bratislava, Slovakia; ^4^1st Department of Otorhinolaryngology, Faculty of Medicine, Comenius University, 85107 Bratislava, Slovakia

## Abstract

TREM-1 and TREM-2 molecules are members of the TREM transmembrane glycoproteins. In our previous study we identified increased expressions of TREM-1 and TREM-2 receptors in pulmonary sarcoidosis (PS). Only a few studies concerning the association between vitamin D and TREM receptor expression can be found. The aim of our current study was to determine the association between the levels of an inactive form of 25(OH)D vitamin and TREM-1 and TREM-2 receptor expressions. We have detected low levels of 25(OH)D vitamin in 79% of PS patients. Only 21% of patients had normal serum level of 25(OH)D vitamin with values clustered within the low-normal range. The most striking findings were the increased TREM-2 expressions on myeloid cells surfaces in BALF of PS patients with normal 25(OH)D vitamin serum levels compared with those with its decreased levels. The total number of TREM-2 positive cells was 5.7 times higher and the percentage of TREM-2 positive cells was also significantly increased in BALF of PS patients with normal compared to PS patients with low 25(OH)D vitamin serum levels. A significant correlation between total TREM-2 expression and vitamin D levels has been detected too. However, we have not detected similar differences in TREM-1expression and 25(OH)D vitamin serum levels.

## 1. Introduction

Sarcoidosis is a granulomatous inflammatory disease of unknown etiology. The antigen responsible for the induction of immune response in sarcoidosis is not yet known. Most evidence supports that the disease is caused by improper, inadequate immune response (dysregulation, hypersensitivity) to an unknown antigen inhaled into the lungs. Different antigens may induce an immune response associated with similar clinical symptoms in genetically predisposed individuals [1, 2]. The immune response can be subsequently modified by neural, hormonal, or environmental factors. Dysregulation of the immune response may also result from vitamin D deficiency as vitamin D plays an important role in immune homeostasis maintaining. Vitamin D receptors may be found on the cell surface of macrophages, dendritic cells, and T- and B-lymphocytes [3]. Vitamin D is essential for the differentiation of monocytes to macrophages or dendritic cells and initiation of T cell response [4, 5]. During inflammation, macrophages increase the amount of vitamin D receptors and hydroxylase D1 activity, an enzyme catalysing the conversion of inactive precursor 25-hydroxyvitamin D (25(OH)D) to the active metabolite 1 alpha 25(OH)(2)D(3) (D-1,25). The active form of vitamin D-1,25 is vital for the production of antimicrobial peptides in macrophages. Vitamin D deficiency can lead to poor elimination of microorganisms and their persistence inside the cells and decreased levels of vitamin D in patients with severe sarcoidosis were accompanied by reduced cathelicidin mRNA expression [6, 7]. A study by Liu et al. showed an increased susceptibility to the tuberculous infection with a more severe course in patients with vitamin D deficiency. Similarly, increased susceptibility to sarcoidosis may be caused by vitamin D deficiency [8]. Furthermore, active vitamin D-1,25 synthesis by macrophages in sarcoid granulomas suggests its increased demand in the ongoing immune response. Local synthesis of the active vitamin D-1,25 may be due to primary deficiency of systemic vitamin D levels (both inactive and active vitamin D). The primary vitamin D deficiency could be the reason for the increased incidence of sarcoidosis in the Nordic countries (lack of sunlight) and in African Americans (skin pigment filter) as the active vitamin D-1,25 synthesis depends on the sunlight exposure. Several studies identified normal or elevated levels of the active form of vitamin D-1,25 but low levels of the inactive form of 25(OH)D vitamin in sarcoidosis [7, 9, 10]. In these studies low levels of 25(OH)D vitamin were found in 80% [9] or in 95% [7] of patients, whereas elevated levels of the active D-1,25 were in 11% [9] or in 10% of patients [7]. Moreover, Barna et al. showed significantly lower values of 25(OH)D vitamin in severe sarcoidosis compared to mild disease with no significant difference in the levels of the active form of vitamin D-1,25 [7]. Professor Sharma (emeritus president of the WASOG) summarized these findings in the review article and suggested that low levels of 25(OH)D vitamin might be present before the development of the disease and might participate in the immunopathogenesis of the disease [11].

TREM (triggering receptor expressed on myelocytes) receptors, first described in 2000, are triggering receptors expressed on the surface of myeloid cells. An increased TREM-1 expression on the surface of myeloid cells occurs in the presence of extracellular bacteria, fungi, and their products [12–14]. Increased TREM-1 expression and increased levels of soluble TREM-1 accompany both infectious and noninfectious inflammatory processes [15, 16]. Triggering receptor expressed on myeloid cells-2 (TREM-2) is involved in cell fusion and granuloma formation, the characteristic sign of sarcoidosis [17]. So, these receptors reflect two typical signs of sarcoidosis: (1), inflammation and antimicrobial response (through TREM-1) and (2) phagocytosis and multinucleated cells and granuloma formation (through TREM-2 and DAP-12 signalling). In our previous study we showed an increased expression of both TREM-1 and TREM-2 receptors on the surface of myeloid cells in bronchoalveolar lavage fluid (BALF) in pulmonary sarcoidosis (PS) and increased levels of soluble sTREM-1 in BALF of PS patients [18, 19]. There are a few studies concerning the levels of vitamin D and TREM receptor expressions. Rigo et al. found that TREM-1 mRNA expression was induced up to 12-fold by active vitamin D-1,25 in bronchial epithelial cells [20].

The serum level of 25(OH)D vitamin is the best indicator of overall vitamin D status, because it reflects the total vitamin D from dietary intake and sunlight exposure. Human monocytes have the ability to convert 25(OH)D vitamin to an active form. Both forms of vitamin D dose dependently inhibit LPS-induced IL-6 and TNF-*α* production by human monocytes [21].

The aim of our current study was to determine the association between the serum levels of an inactive form of 25(OH)D vitamin and TREM-1 and TREM-2 receptor expressions. We want to find out which of these TREM receptors (and indirectly, inflammation versus phagocytosis and multinucleated giant cells and granuloma formation) is mostly influenced by vitamin D serum concentration. No such study in sarcoidosis patients has been published yet.

## 2. Patients and Methods

43 patients with pulmonary sarcoidosis (PS) were prospectively enrolled in the study. This study was approved by the Local Ethical Committee of Faculty of Medicine Comenius University in Bratislava and written informed consent was obtained from all patients.

The diagnosis of PS was established in compliance with criteria recommended by the American Thoracic Society (ATS), the European Respiratory Society (ERS), and the World Association of Sarcoidosis and Other Granulomatous Disorders (WASOG): typical clinical-radiological presentation, the histological evidence of noncaseating granuloma, and/or an increased CD4/CD8 ratio (>3.5) in BALF [22]. Some patients refused biopsy, because they did not feel so ill to undergo an invasive diagnostic procedure. According to chest radiographic staging, 8 PS patients had stage I of the disease, 30 patients had stage II, three patients had stage III, and two patients had stage IV of the disease (Table 1). None of the patients received corticosteroid treatment before BAL procedure. Other possible diseases causing granuloma were excluded.

All patients in our study had no comorbidities, negative BALF culture (aerobic and anaerobic bacteria, yeasts,* Mycobacterium tuberculosis* culture), and negative serology results (for Chlamydia, Mycoplasma, Aspergillus).

BAL procedure during bronchoscopy was carried out at the Department of Pneumology and Phthisiology, Faculty of Medicine, Comenius University in Bratislava. BALF harvesting was performed by instillation of 100 mL of sterile normal saline (in two successive 50 mL aliquots) into the right middle lobe and aspirated by gentle suction using a flexible fiberoptic bronchoscope.

BALF was first filtered through a double layer of sterile gauze and centrifuged at 300 g for 15 min at 4°C, supernatants were collected, and finally, the pelleted cells were gently resuspended in PBS (phosphate buffer saline) and used for flow cytometric staining.

TREM-1 and TREM-2 expression was examined by flow cytometry on cells in freshly isolated BALF (10–20 mL) samples. For the flow cytometric analysis of BALF cells, specimens were double stained with a phycoerythrin- (PE-) conjugated mouse anti-human TREM-1 (clone 193015) and rat allophycocyanin- (APC-) conjugated anti-human TREM-2 (clone 237920) monoclonal antibodies (R&D Systems, Minneapolis, MN, USA). An anti-human/mouse IgG1 K isotype control-phycoerythrin conjugated (eBioscience, Inc.) and anti-human/rat IgG2b K isotype control-allophycocyanin conjugated (eBioscience, Inc.) monoclonal immunoglobulins were used. Moreover, staining with fluorescein isothiocyanate- (FITC-) conjugated mouse anti-human CD14 (Beckman Coulter Company, Immunotech, Marseille, France) was performed to detect monocyte/macrophages and granulocytes, respectively. Cells were analysed on a NAVIOS (Beckman Coulter, Inc, USA) cytometer using CXP software (Beckman Coulter, Inc, USA). TREM-1 and TREM-2 expressions were measured as the total number and the percentage of TREM-1 and TREM-2 positive cells out of all CD14^+^ cells and mean fluorescence intensity (MFI) of 10,000 cells in BALF subtracted from isotype control. Total number of TREM-1 and TREM-2 positive cells was calculated from absolute count of BALF cells evaluated by flow cytometer. The absolute count of cells per milliliter was obtained of filtered, not concentrated, and not centrifuged BALF by Flow-Count Fluor spheres (Beckman Coulter Inc.).

The serum levels of 25(OH)D vitamin and its levels in BALF were tested by high-performance liquid chromatography (HPLC) in a specialized biochemical laboratory Medirex Ltd. in Bratislava.

## 3. Statistical Analysis

The one-sample Kolmogorov-Smirnov test was used to determine whether the investigated population followed a normal distribution. Either nonparametric Mann-Whitney *U*-test or parametric unpaired *t*-test with Welch correction was used to determine the difference and the statistical significance. Pearson or Spearman test was used to determine data correlation. We used min-max data normalization formula for variables: total number, percentage, and SFI.

## 4. Results

### 4.1. Serum Levels of 25(OH)D Vitamin in Patients with Pulmonary Sarcoidosis

Normal serum levels of 25(OH)D vitamin (30–50 ng/mL) were found only in 21% of patients with pulmonary sarcoidosis and these values were clustered within the low-normal range in all patients (mean: 33.1 ng/mL; max: 35.3 ng/mL). 79% of PS patients had low serum levels of 25(OH)D vitamin (Table 2). The concentration of 25(OH)D vitamin in BALF was in all PS patients under the detection limit (<2.70 ng/mL).

### 4.2. Comparison of TREM Receptor Expressions in BALF of PS Patients with Normal versus Low 25(OH)D Vitamin Serum Levels

The expression of TREM receptors is defined by three variables (absolute number of TREM positive cells, percentage of TREM positive cells, and the mean fluorescence intensity (MFI)). First, we compared each variable separately: total number (Tables 3 and 4, Item (1)), percentage (Tables 3 and 4, Item (2)), and mean fluorescence intensity (Tables 3 and 4, Item (3)).

Secondly, we determined the total expression taking into account all three variables (total number, percentage, and MFI). Considering that each variable was in different value scale prevented the direct addition; these data (total number, percentage, and MFI) were transformed using min-max normalization method. Total TREM expression was then calculated as a sum of all transformed data (all variables expressed as comparable numbers). In addition, we specified the total expression as a count of positive variables (total number, percentage, and MFI) in individual patients and assigned 0 to be no positive variable, 1 to be one positive variable, 2 to be two positive variables, and 3 to be three positive variables.

Finally, we calculated differences in TREM receptor expressions in patients with normal and decreased 25(OH)D vitamin serum levels, the total of 5 variables:

- Measured Values:
  1. Total number (Tables 3 and 4, Item (1)) of TREM positive cells.
  2. Percentage (Tables 3 and 4, Item (2)) of TREM positive cells.
  3. Mean fluorescence intensity (Tables 3 and 4, Item (3)) of TREM-positive cells.
- Total expression of all three variables:
  (4) Sum of variables expressed in a scale 0-1 using min-max normalization method (Tables 3 and 4, Item (4)).
  (5) Number of positive variables determining TREM score (Tables 3 and 4, Item (5)).

### 4.3. Comparison of TREM-1 Expressions on Myeloid Cell Surfaces in BALF of PS Patients with Normal versus Low 25(OH)D Vitamin Serum Levels

We did not find statistically significant differences in TREM-1 expression in patients with normal 25(OH)D vitamin compared with patients with low 25(OH)D vitamin levels; conversely the *P* value approached 1, suggesting more for independence between serum 25(OH)D vitamin and TREM-1 expression (Table 3).

### 4.4. Comparison of TREM-2 Expressions on the Surface of Myeloid Cells in BALF of PS Patients with Normal versus Low 25(OH)D Vitamin Serum Levels

In contrast to TREM-1, we identified significant differences in TREM-2 expressions on myeloid cell surfaces in BALF of pulmonary sarcoidosis patients. The total number, percentage proportion, and total expression of TREM-2 were higher in patients with normal 25(OH)D vitamin levels compared to patients with decreased 25(OH)D vitamin levels and the differences were statistically significant (Table 4). The most significant differences were detected in the total TREM-2 expression (Table 4; Item (4) and (5)).

### 4.5. Correlation between TREM Expressions in BALF and Serum 25(OH)D Vitamin Levels in Pulmonary Sarcoidosis Patients

We evaluated the correlation of TREM receptor expressions and serum 25(OH)D vitamin levels.

### 4.6. Correlation between TREM-1 Expression on Myeloid Cell Surfaces in BALF in PS Patients and Their 25(OH)D Vitamin Serum Levels

No statistically significant correlation in any variables assessing the TREM-1 expressions was detected (Table 5).

### 4.7. Correlation between TREM-2 Expression on Myeloid Cell Surfaces in BALF of PS Patients and Their 25(OH)D Vitamin Serum Levels

We did not show a statistically significant correlation in total number, percentage, and MFI of TREM-1 on the surface of myeloid cells in BALF of PS patients and their 25(OH)D vitamin serum levels. However, we found a statistically significant correlation between the total expression of TREM-2 and the serum levels of 25(OH)D vitamin (Table 6; Item (4)).

## 5. Discussion

TREM-1 and TREM-2 molecules were discovered as members of the TREM transmembrane glycoproteins belonging to the single immunoglobulin variable (IgV) domain receptor family. As the cytoplasmic tails of both TREM-1 and TREM-2 lack signalling motifs, the signalling cascade via ITAM motif of DAP12 is required [23–25]. While TREM-1/DAP12 signalization has proinflammatory activity, strikingly, the TREM-2/DAP-12 pathway may provide both inhibitory and activating signals depending on their microenvironment [24, 25]. Moreover, the biological functions of TREM-2/DAP12 pathway may depend on the influence of additional receptors (TLRs) and on the presence of TREM-2 ligands, different cytokines (IL-4, IL-13, IFN-*γ*), and *β*-integrins, which may finally induce activating or inhibitory signals through TREM-2/DAP-12 pathway [24, 26, 27].

TREM-2 is expressed on osteoclasts, microglial cells, dendritic cells (DCs), and tissue infiltrating macrophages from the circulation (not resident macrophages) and macrophages activated by IL-4 and IL-13 [26]. Engagement of TREM-2/DAP-12 under inflammatory conditions has been suggested to promote differentiation of macrophages, DCs, and microglial cells. Human TREM-2 regulates the activation of DCs, macrophages, osteoclasts, and microglial cells and plays a critical role in fine-tuning inflammatory response [26–29]. TREM-2 is a key negative regulator of autoimmunity, inhibits the production of TNF and IL-6 by macrophages, and is responsible for DAP12 mediated inhibition of inflammatory response induced by TLR ligands. TREM-2/DAP12 signalisation triggers the macrophage differentiation and their programming into fusogenic state [30]. TREM-2/DAP12 also play a positive role in phagocytosis, binding both exogenous and endogenous ligands. TREM-2 expression on both microglia and macrophages is associated with specific activated cell phenotype, which performs important protective functions, such as tissue repair, control of local inflammation, or phagocytosis of dying cells. TREM-2 can also bind several types of fungi and bacteria and in association with DAP12 can promote their phagocytosis [31–34].

A link between elevated vitamin D levels and sarcoidosis was proposed over 50 years ago. Granulomas in pulmonary sarcoidosis have the ability to form an active form of vitamin D and subsequent hypercalcemia may lead to renal failure [35]. In our work from 2013 we identified increased expressions of TREM-1 and TREM-2 receptors on the surface of myeloid cells and increased levels of soluble TREM-1 in bronchoalveolar lavage fluid in pulmonary sarcoidosis patients [18, 19].

The aim of our current study was to determine the association between the serum levels of an inactive form of 25(OH)D vitamin and TREM-1 and TREM-2 receptor expressions, which undoubtedly participate in the immune response accompanying sarcoidosis. In our work we detected low levels of 25(OH)D vitamin in 79% of patients with pulmonary sarcoidosis. Only 21% of patients had normal serum levels of 25(OH)D vitamin and these values were clustered within the low normal range. Our findings are in agreement with other studies [7, 9]. The concentrations of 25(OH)D vitamin in BALF of all PS patients were under the detection limit. The most striking result was the increased TREM-2 expressions on the surfaces of myeloid cells in BALF of PS patients with normal serum levels of 25(OH)D vitamin compared to patients with decreased 25(OH)D vitamin levels. The total number of TREM-2^+^ cells was 5.7 times higher and the percentage of TREM-2 positive cells was also significantly increased in BALF of PS patients with normal 25(OH)D vitamin level comparing to PS patients with low vitamin D serum levels. A significant correlation between total TREM-2 expression in BALF and 25(OH)D vitamin levels was detected too. However, we did not prove similar differences in TREM-1 expression and 25(OH)D vitamin serum levels.

There is not any theory explaining these findings. Only a few studies (4-5) analysed the influence of vitamin D to TREM receptor expressions; however, no experiments concerning the effect of vitamin D to TREM-2 expression on macrophages have been done [20, 36–38]. None of these studies concerned sarcoidosis and all were performed by active form of vitamin D (1,25(OH)2D3) and most of them “*in vitro.*” So we cannot compare directly our results with findings of other authors. Which concerns TREM-1, Kim et al. showed that 1,25(OH)2D3, an active form of vitamin D, upregulating TREM-1 expression (TREM-1 mRNA), may function as an enhancer of immune response in addition to inducing the antimicrobial peptide cathelicidin (LL-37 mRNA) [37]. Other authors from the same group suggest that expression of TREM-1 is induced through the mTOR signalling pathway in human macrophages [38]. There is a possibility that increased TREM-2 expression and vitamin D-dependent antimicrobial mechanisms may be two parts of the immune response in sarcoidosis, in which the vitamin D level and TREM-2 expression somehow interact with each other. Our result points to the role of vitamin D in increasing the TREM-2 expressions. It is known that TREM-2 potentiates granuloma formation and subsequently, macrophages inside of granuloma enhance the production of active vitamin D. The biological activity of vitamin D and the consequences of TREM-2 activation meet each other in some points. The immune response in sarcoidosis is defined by pathogenic properties of antigen (microorganisms or noninfectious stimuli). Possible explanation might be as follows: antigen elimination may require vitamin D-dependent antimicrobial mechanisms (e.g., cathelicidin production) [7] but also TREM-2/DAP12 pathway for activation of phagocytosis. Vitamin D deficiency allows microbes to survive inside the cells and due to a transient breakdown of immunological surveillance, microbial invader could exploit the monocyte differentiation pathway to DCs to its own advantage [37, 38]. For example, the latest studies show that* Candida albicans* and* Mycobacterium tuberculosis *(both potential etiologic agents) affecting the membrane molecule expressions interfere with fully active DCs generation [37, 38]. The DCs differentiation is potentiated also by activation of TREM-2/DAP12 pathway; moreover, this process is associated with the differentiation of macrophages to their fusogenic state and creation of large multinucleated giant cells typical for granulomas in sarcoidosis [24, 26, 30]. And granulomas in pulmonary sarcoidosis are known to form an active form of vitamin D [22]. Moreover, TREM-2 activation leads to decreased proinflammatory cytokine production, which subsequently may facilitate the survival of microorganisms; however the granuloma formation might be responsible for at least the encirclement of pathogen in the state of inadequate immunity.

From work of Kim et al. [37] we know that “*in vitro*” physiological concentrations of active vitamin D induce expression of TREM-1 mRNA and cathelicidin mRNA (antimicrobial factor). In our study, PS patients with normal levels of inactive vitamin D (values clustered within the low normal range) are probably insufficient for production of cathelicidin and TREM-1 expression (this is a priori enhanced in PS patients) but however are sufficient for potentiation of phagocytosis and granuloma formation what in cases of inadequate immunity. The killing process that precedes phagocytosis needs probably higher vitamin D levels. Further studies are needed to clarify these findings.

## 6. Conclusion

PS patients have increased expressions of both TREM-1 and TREM-2 on myeloid cells surfaces in BALF of PS patients. In our present study we detected significantly increased total number and percentage of TREM-2 positive cells in BALF of PS patients with normal serum levels of 25(OH)D vitamin compared with PS patients with low vitamin D levels. Significant correlation between total TREM-2 expression and 25(OH)D vitamin serum level was also detected.

## Figures and Tables

**Table 1:** Characteristics of study subjects.

	PS
Number of patients	43
Male (%)	58.00
Mean age	39
DLCO (mean ± SD)	69.5 ± 15.7
Chest X-ray stage, (PS, stage I/II/III/IV)	8/30/3/2
>20% Lymphocytes in BALF (% patients)	73%
CD4/CD8 >3,5 (% patients)	67%
Lymphadenopathy (% patients)	88%
Granuloma proven (biopsy)	28%

PS: pulmonary sarcoidosis; DLCO: percentage of predicted carbon monoxide lung diffusion capacity.

**Table 2:** Serum levels of 25(OH)D vitamin in patients with pulmonary sarcoidosis.

	Normal levels of 25(OH)D vitamin (ng/mL)	Low levels of 25(OH)D vitamin (ng/mL)
Percentage of patients	21	79
Mean	33.1	17.0
Range	30.3–35.3	3.9–29.4

**Table 3:** Comparison of TREM-1 expressions on the surface of myeloid cells in BALF of patients with normal versus low 25(OH)D vitamin serum levels.

TREM-1	Normal 25(OH)D levels	Low 25(OH)D levels	*P*
(1) Total No.	Mean ± SD: 316.6 ± 683.1 Median: 23.1 Range: 11.3–2083.2	Mean ± SD: 72.8 ± 82.3 Median: 43.0 Range: 0.48–361.6	0.7358^*∗*^

(2) Percentage	Mean ± SD: 48.5 ± 25.0 Median: 58.1 Range: 9.9–74.7	Mean ± SD: 48.6 ± 22.3 Median: 48.0 Range: 1.5–86.2	0.9872^*∗∗*^

(3) MFI	Mean ± SD: 6.7 ± 3.3 Median: 6.0 Range: 1.5–12.2	Mean ± SD: 7.9 ± 6.1 Median: 5.9 Range: 1.3–27.5	0.8719^*∗*^

(4) Total expression (min-max data transformation)	Mean ± SD: 1.13 ± 1.09 Median: 0.84 Range: 0.21–3.83	Mean ± SD: 0.82 ± 0.41 Median: 0.85 Range: 0.07–2.05	0.7358^*∗*^

(5) Total expression (No. of positive variables)	Mean ± SD: 1.0 ± 0.9 Median: 1 Range: 0–2	Mean ± SD: 0.9 ± 0.8 Median: 1 Range: 0–3	0.6297^*∗*^

^*∗*^Mann-Whitney test, ^*∗∗*^ *t*-test.

**Table 4:** Comparison of TREM-2 expressions on the surface of myeloid cells in BALF of PS patients with normal versus low 25(OH)D vitamin serum levels.

TREM-2	Normal 25(OH)D levels	Low 25(OH)D	*P*
(1) Total number	Mean ± SD: 188.82 ± 396.2 Median: 60.71 Range: 5.99–1240.9	Mean ± SD: 32.78 ± 22.8 Median: 26.09 1.74–104.8	0.0131^*∗*^

(2) Percentage	Mean ± SD: 48.74 ± 18.0 Median: 48.5 Range: 15.1–82.3	Mean ± SD: 31.6 ± 16.8 Median: 30.2 Range: 2.7–87.4	0.0071^*∗∗*^

(3) MFI	Mean ± SD: 14.2 ± 15.2 Median: 4.5 Range: 2.28–44.8	Mean ± SD: 9.8 ± 16.1 Median: 4.5 Range: 0.79–74.3	0.2680^*∗*^

(4) Total expression (min-max data transformation)	Mean ± SD: 4.34 ± 0.77 Median: 4.33 Range: 2.8–5.2	Mean ± SD: 3.52 ± 0.88 Median: 3.62 Range: 0.68–5.6	0.0039^*∗*^

(5) Total expression (number of positive variables)	Mean ± SD: 1.89 ± 0.78 Median: 2 Range: 0–3	Mean ± SD: 0.62 ± 0.70 Median: 1 Range: 0–3	0.0005^*∗*^

^*∗*^Mann-Whitney test, ^*∗∗*^ *t*-test.

**Table 5:** Correlation of TREM-1 expressions on the myeloid cell surfaces in BALF of PS patients and their 25(OH)D vitamin serum levels.

Correlation of TREM-1 expression and vitamin D-25 levels	(1) Total number of TREM-1 positive cells	(2) Percentage of TREM-1 positive cells	(3) MFI	(4) Total expression (min-max data transformation)
*P*	0.7481^*∗*^	0.3876^*∗∗*^	0.5414^*∗*^	0.1298^*∗*^
*R*	0.05044	0.1351	0.09574	0.2347
95% CI	−0.2624–0.3537	−0.1723–0.4185	−0.2195–0.3929	−0.0798–0.5067

MFI: mean fluorescence intensity, Total expression: total expression as the sum of variables after min-max data normalization; ^*∗*^Spearman's correlation, ^*∗∗*^Pearson's correlation.

**Table 6:** Correlation of TREM-2 expressions on myeloid cell surfaces in BALF of PS patients and the 25(OH)D vitamin serum levels.

Correlation of TREM-2 expressions and serum D-25 levels	(1) Total number	(2) Percentage	(3) MFI	(4) Total expression (min-max data transformation)
*P*	0.4277^*∗*^	0.0622^*∗∗*^	0.3801^*∗*^	0.0457^*∗*^
*R*	0.1241	0.2742	0.1373	0.3064
95% CI	−0.1929–0.4169	−0.01417–0.5204	−0.1790–0.4279	−0.002567–0.562

Total expression: total expression as the sum of variables after min-max data normalization; ^*∗*^Spearman's correlation, ^*∗∗*^Pearson's correlation.
